# Production of Added-Value Chemical Compounds through Bioconversions of Olive-Mill Wastewaters Blended with Crude Glycerol by a *Yarrowia lipolytica* Strain

**DOI:** 10.3390/molecules24020222

**Published:** 2019-01-09

**Authors:** Dimitris Sarris, Anna Rapti, Nikolaos Papafotis, Apostolis A. Koutinas, Seraphim Papanikolaou

**Affiliations:** 1 Department of Food Science & Human Nutrition, Agricultural University of Athens, 11855 Athens, Greece; nick.papafotis@gmail.com (N.P.); akoutinas@aua.gr (A.A.K.); 2 Department of Food Science & Nutrition, School of the Environment, University of the Aegean, 81400 Myrina, Lemnos, Greece; anna.rapti@outlook.com

**Keywords:** olive mill wastewaters, *Yarrowia lipolytica*, polyols, cellular lipids, citric acid, crude glycerol

## Abstract

Olive mill wastewaters (OMW) are the major effluent deriving from olive oil production and are considered as one of the most challenging agro-industrial wastes to treat. Crude glycerol is the main by-product of alcoholic beverage and oleochemical production activities including biodiesel production. The tremendous quantities of glycerol produced worldwide represent a serious environmental challenge. The aim of this study was to assess the ability of *Yarrowia lipolytica* strain ACA-DC 5029 to grow on nitrogen-limited submerged shake-flask cultures, in crude glycerol and OMW blends as well as in media with high initial glycerol concentration and produce biomass, cellular lipids, citric acid and polyols. The rationale of using such blends was the dilution of concentrated glycerol by OMW to (partially or fully) replace process tap water with a wastewater stream. The strain presented satisfactory growth in blends; citric acid production was not affected by OMW addition (Cit_max_~37.0 g/L, Y_Cit/Glol_~0.55 g/g) and microbial oil accumulation raised proportionally to OMW addition (L_max_~2.0 g/L, Y_L/X_~20% *w*/*w*). Partial removal of color (~30%) and phenolic compounds (~10% *w*/*w*) of the blended media occurred. In media with high glycerol concentration, a shift towards erythritol production was noted (Ery_max_~66.0 g/L, Y_Ery/Glol_~0.39 g/g) simultaneously with high amounts of produced citric acid (Cit_max_~79.0 g/L, Y_Cit/Glol_~0.46 g/g). Fatty acid analysis of microbial lipids demonstrated that OMW addition in blended media and in excess carbon media with high glycerol concentration favored oleic acid production.

## 1. Introduction

Olive mill wastewaters (OMW) are the major effluent deriving from the industrial production of olive oil. It is considered as one of the most challenging agro-industrial wastes to treat [1]. OMW has dark color, strong odor and (phyto-)toxic properties, mainly attributed to high quantities of phenolic compounds that it contains [2,3,4,5,6]. OMW is produced in vast volumes, seasonally and geographically scattered [7] and is characterized by extremely high biological (BOD) and chemical oxygen demand (COD) (values up to 200–400 times higher than typical municipal sewage) [1]. Despite the fact that this effluent constitutes a serious environmental threat, it is commonly discarded directly to the environment (agricultural land, aquatic environment, artificial lagoons) without any prior treatment [8]. Therefore, the development of an effective and viable treatment technology for this waste stream remains a priority. Various physico-chemical treatment processes are reported in literature [9,10,11] but in most cases they are limited to small-scale operations. Besides its phenolic content, the OMW organic fraction is also composed of sugars, organic acids and residual oil [10,12]. Due to its assimilable carbon sources content, OMW could be considered as a valuable substrate for biotechnological applications (used either as process water or as a nutrient-rich medium). OMW-based media have been used as a growth substrate for a plethora of microorganisms resulting in the production of metabolic compounds such as biomass and single cell oil [1,8,12,13,14,15], citric acid [5,13,15,16], methane and polymers [12,17,18], enzymes [4,5,12,15], exopolysaccharides [12] and bioethanol [7,19,20,21].

Crude glycerol is the main by-product of biodiesel production. Biodiesel, (produced by transesterification of triglycerides with alcohol, also called alcoholysis), is a renewable source of energy and its production utilizes vegetable oils, animal fats or waste cooking oils and fats as raw materials [22,23,24,25,26,27]. The production process of this biofuel results in ~10% (*w*/*w*) of crude glycerol as by-product [24,27,28]. Crude glycerol generation as a waste stream is not only limited to biodiesel industry but extends to other oleochemical activities (i.e., fat saponification) that employ transformation of vegetable or animal fats (resulting in liquid waste stream containing high levels of ~55–90% *w*/*v* of glycerol) [29,30]. Moreover, indicative volumes of waters containing glycerol may derive from the food industry and specifically from alcoholic beverage production units. When ethanol is separated via distillation (after fermentation process and bioethanol production), the remaining liquid fraction contains ~2% *w*/*v* glycerol [30]. To this end, crude glycerol could be considered as an alternative carbon source rather than a waste. It could be used as a valuable substrate for microorganisms, with various applications in industry [31], since a plethora of microbial compounds (such as 1,3-propanediol, hydrogen, propanoic acid, trehalose, single cell oil, *n*-butanol, glyceric acid, citric acid, ethanol, polyunsaturated fatty acids, biopolymers) can be produced [23,32]. The rationale behind the use of crude glycerol and OMW blends as substrate is to partially or fully replace process tap water in concentrated glycerol dilution by utilizing a wastewater stream. OMW has been previously used in blends with molasses and pure glycerol [19,33] or in OMW-based media (i.e., fortified with commercial glucose) [8,13,16,20,33,34].

Citric acid is a compound that finds numerous applications as an acidulant, flavoring agent and antioxidant within beverages and confectionery, infant formulas, the chemical and pharmaceutical industries [26,35]. Citric acid is recognized as safe, has a pleasant acid taste, and high water solubility as well as chelating and buffering properties [36]. Polyols (sugar alcohols) are carbohydrates with a carbonyl group reduced to a corresponding hydroxyl group, presenting unique nutritional (non-cariogenic and less calorigenic properties as also with low insulin-mediated response) and other properties (as precursor compounds for polyurethanes, resins, surfactants and intermediates towards hydrocarbons production), thus they are used as nutraceuticals and pharmaceuticals in medical and chemical industries [37,38,39,40,41,42]. Microbial (cellular) lipids are (edible) lipophilic compounds produced by various microorganisms; they often possess special composition and structure and as such, they are of great interest for food and pharmaceutical industries [43,44].

The aim of the present study was to assess the ability of *Y. lipolytica* strain ACA-DC 5029 to grow on blends of crude glycerol and OMW (constant initial glycerol concentration and increasing volumes of OMW in the media) and produce (high-) added value compounds. Citric acid, cellular lipid and polyols production was monitored and analyzed. Additional experiments including media with high initial crude glycerol concentrations were conducted. The rationale of such experiments was to evaluate routes for the valorization of large amounts of glycerol and potentially enhance the production of yeast metabolites. To the knowledge of the authors, this is the first report in the international literature in which blends of OMW and crude biodiesel-derived glycerol are used as substrate for (high-) added value compounds by yeasts. Such a fact may be of great importance since in a potential process scale-up (application in large-scale bioprocesses) OMW could partially or, in some cases, totally substitute tap water for glycerol dilution, thus offering an attractive alternative. In terms of waste bio-remediation, an additional aim of this study was to simultaneously reduce the color and phenolic compound concentration of the media (appearing due to OMW presence) via the proposed bioprocess.

## 2. Results

Initially, the kinetic behavior of *Y. lipolytica* strain ACA-DC 5029 grown on media with blends of OMW and crude glycerol was studied. Various volumes of OMW and tap water were added in the blended media, resulting in initial concentration of phenolic compounds (Ph_0_) of ~1.0 g/L, ~2.0 g/L and ~3.5 g/L. The initial concentration of crude glycerol remained constant at ~70 g/L. Control trials (no OMW addition) were also performed, as the addition of phenolic compounds could act upon the physiological and kinetic behavior of the yeast. The ability of the yeast strain to grow on such blends and produce citric acid, polyols (mannitol, arabitol, erythritol), biomass and cellular lipid was evaluated in submerged shake-flask nitrogen limited cultures (as nitrogen depletion is essential in order the above-mentioned compounds to be synthesized [45,46]). The obtained results are illustrated in Table 1 and Table 2. Furthermore, additional experiments (submerged shake-flask nitrogen limited cultures) including media (no OMW addition) with high initial crude glycerol concentration of ~120 g/L and of ~170 g/L were conducted. The obtained results are illustrated in Table 3 and Table 4.

### 2.1. Growth of Y. lipolytica Strain ACA-DC 5029 in Media Containing Blends of OMW and Crude Glycerol with Initial Concentration of ~70 g/L

#### 2.1.1. Biomass and Intra-Cellular Polysaccharides (IPS) Evolution

*Y. lipolytica* strain ACA-DC 5029 presented satisfactory growth in both control (no OMW addition) and blended media. In all cases, the applied substrate was totally assimilated. Maximum biomass production (X*max*) ranged between 8.72–11.96 g/L, with the respective yield of biomass per glycerol consumed (Y_X/Glol_) in the range of 0.13–0.18 g/g (Table 1). Overall, the maximum biomass production was noted in control trials reaching 11.96 g/L (Y_X/Glol_ = 0.17 g/g). With regards to trials with blended media (initial phenolic compound concentration of ~1.0 g/L and ~2.0 g/L), biomass production was not significantly different compared to control experiments. Specifically, in cultures with Ph_0_~1.0 g/L, maximum biomass production reached 11.56 g/L (Y_X/Glol_ = 0.18 g/g) and in cultures with Ph_0_~2.0 g/L, maximum biomass production reached 11.58 g/L (Y_X/Glol_ = 0.17 g/g). On the other hand, when maximum OMW volume was added to the media, resulting in initial phenolic compound concentration of ~3.5 g/L, cellular production was notably reduced (X*max* = 8.72 g/L; Y_X/Glol_ = 0.13 g/g) compared to both control and blended cultures (Table 1). This fact suggested that the addition of phenolic compounds (due to OMW presence) into the media seems to obstruct biomass production.

**Figure 1:**
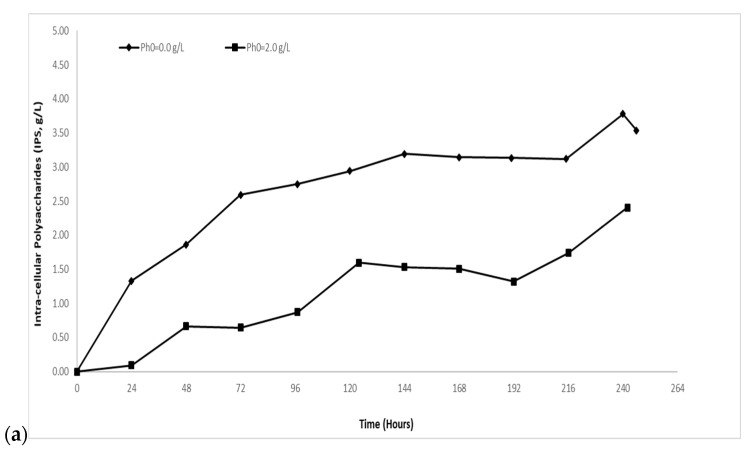

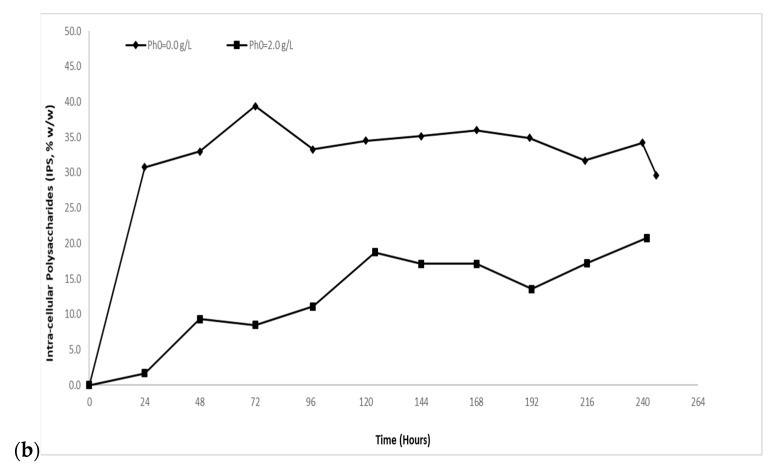
(**a**) Intra-cellular polysaccharides (IPS, g/L) and (**b**) yield of intra-cellular polysaccharides produced per biomass produced (% *w*/*w*) evolution during growth of *Yarrowia lipolytica* strain ACA-DC 5029 on media with ~70 g/L initial crude glycerol concentration (no OMW addition) (-♦-) and on media with ~70 g/L initial crude glycerol concertation, blended with olive mill wastewaters offering initial phenolic compounds concentration of ~2.0 g/L (-■-). Culture conditions as described in Table 1. Each point is the mean value of two independent measurements.

The accumulation of intra-cellular polysaccharides (IPS) was monitored throughout all stages of the cultures. Maximum IPS concentration observed was 3.78 g/L with subsequent yield in dry weight 34.2% *w*/*w* in the control experiment (no OMW addition; Figure 1a,b). Endo-polysaccharide formation was gradually increased up to ~34.0% *w*/*w* until 48 h after inoculation and seemed to remain constant at ~34.0–39.0% *w*/*w* until the end of the fermentation (Figure 1b). Comparing the control experiment with the trials using blended media, it should be noted that OMW addition negatively affected the accumulation of IPS (Figure 1a,b). On the other hand, the IPS production in blended cultures with increasing phenolic compound concentration (OMW added in different volumes) remained largely unchanged; in Ph_0_~1.0 g/L, maximum IPS concentration was 2.37 g/L with subsequent yield in dry weight 20.1% *w*/*w*, in Ph_0_~2.0 g/L, IPS_max_ = 2.40 g/L and yield in dry weight 20.8% *w*/*w* and in Ph_0_~3.5 g/L, IPS_max_ = 2.01 g/L and yield in dry weight 24.2% *w*/*w*. With regards to blended media, endopolysaccharide formation gradually increased up to ~20.0% *w*/*w* (compared to control experiments) until 96–120 h after inoculation and remained constant at ~20.0–24.0% *w*/*w* until the end of fermentation.

#### 2.1.2. Citric Acid and Polyols Production

In the control experiment (no OMW addition), citric acid secretion by the strain reached a maximum value (Cit_max_) of 42.5 g/L with the respective yield of citric acid per glycerol consumed (Y_Cit/Glol_) 0.59 g/g. The addition of OMW in the media affected negatively citric acid production, compared to control trial. Specifically, in cultures with initial phenolic compound concentration of ~1.0 g/L, Cit_max_ was 28.8 g/L (Y_Cit/Glol_ = 0.41 g/g), reaching 31.5 g/L (Y_Cit/Glol_ = 0.45 g/g) at Ph_0_ = 2.0 g/L and 37.4 g/L (Y_Cit/Glol_ = 0.55 g/g) in OMW-based media with Ph_0_ = 3.5 g/L. On the other hand, by comparing blended media only, it seems that OMW addition favors the accumulation of citric acid (Table 1). Thus, media including crude glycerol and OMW (no tap water added) could be considered more suitable for citric acid production.

The *Y. lipolytica* ACA-DC 5029 strain presented the ability to produce the polyols mannitol, arabitol and erythritol when crude glycerol was used as substrate, in both control and OMW-blended cultures. The maximum values of produced mannitol (Man_max_) were in the range of 5.3 g/L and 13.1 g/L, with respective values of yield of mannitol per glycerol consumed (Y_Man/Glol_) in the range of 0.08 g/g and 0.21 g/g (Table 1). Compared with control trials, the addition of OMW did not affect mannitol production, except for the experiment with initial phenolic compound concentration of ~2.0 g/L, where Man_max_ reached 5.3 g/L (Y_Man/Glol_ = 0.45 g/g). A similar trend occurred in arabitol production, as it was not majorly affected by the addition of OMW (Ara_max_ = 2.0–3.2 g/L, Y_Ara/Glol_ = 0.03–0.05 g/g) (Table 1). On the other hand, erythritol production was notably reduced when OMW was added into the media, compared to control cultures. Specifically, the maximum value of produced erythritol (Ery_max_) in control cultures was 14.9 g/L, with the yield of erythritol per glycerol consumed (Y_Ery/Glol_) equal 0.23 g/g. In blended media with initial phenolic compound concentration of ~1.0 g/L, the value of Ery_max_ was 8.9 g/L (Y_Ery/Glol_ = 0.19 g/g). In blended media with initial phenolic compound concentration of ~3.5 g/L, erythritol production was highly affected (Ery_max_ = 2.4 g/L, Y_Ery/Glol_ = 0.04 g/g) (Table 1). These observations indicated a shift of the microbial metabolism towards citric acid production at the expense of mainly erythritol production, due to the addition of OMW into the medium. The only exception from this trend was the trial with initial phenolic compound concentration of ~2.0 g/L, where the value of Ery_max_ was 13.5 (Y_Ery/Glol_ = 0.19 g/g).

#### 2.1.3. Biogenesis of Cellular lipids and Fatty Acid (FA) Composition

Total microbial lipids were extracted from dry cells in all growth phases in all media (control and blended). The maximum values (L_max_) were in the range of 1.27 and 1.32 g/L with total lipid in dry biomass (Y_L/X_) values of 10.6–20.0% *w*/*w* (Table 1). As reported in previous studies, various *Y. lipolytica* strains do not demonstrate the typical oleaginous character during growth on batch glycerol-based nitrogen-limited experiments [29,43,45,47]. These findings are in accordance with the present study, as it may be assumed that microbial metabolism was shifted towards extra-cellular compounds biosynthesis (citric acid and polyols) rather than cellular lipids. In the control experiment, lipid concentration reached the maximum value (L_max_) of 1.27 g/L with respective yield of lipid per biomass produced (Y_L/X_) equal to 10.6% (*w*/*w*). Surprisingly enough, even though OMW is considered as an inhibition factor mainly due to its inherent presence of phenolic compounds, the addition of OMW into the blended media stimulated total lipid production compared to the control trials (Table 1). Specifically, in cultures with initial phenolic compound concentration of ~1.0 g/L, the value of L_max_ was 1.65 g/L (Y_L/X_ = 17.8% *w*/*w*), in cultures with initial phenolic compound concentration of ~2.0 g/L the value of L_max_ was 1.95 g/L (Y_L/X_ = 20.0% *w*/*w*) and only in cultures with initial phenolic compound concentration of ~3.5 g/L the value of L_max_ was slightly reduced at1.32 g/L (Y_L/X_ = 15.9% *w*/*w*). Such results indicate that OMW may act as a natural “lipogenic” medium and they are in accordance with literature findings, as it has been reported that the presence OMW enhances lipid accumulation not only in *Y. lipolytica* strains [13,34] but also in other genera [8]. 

The fatty acid (FA) composition of the intra-cellular lipids was analyzed at various growth phases for control and blended cultures (Table 2). The principal FAs detected were C16 and C18 aliphatic chains. It should be noted that in all cultures (control and blended) the unsaturation index (UI) of FA was different in various culture phases. In most cases, the UI increased during the stationary growth phase as the concentration of ^Δ9^C16:1, ^Δ9^C18:1 and ^Δ9,12^C18:2 increased, whereas the concentration of C16:0, C18:0 decreased. Compared to control experiments, UI of lipids produced by the yeast in blended media seemed to remain unaffected. The addition of OMW resulted in a significant increase in the concentration of ^Δ9^C18:1 (in the blended cultures with Ph_o_~2.0 g/L the concentration of ^Δ9^C18:1 reached ~60% *w*/*w*) and in a slight increase of ^Δ9,12^C18:2 concentration (Table 2). On the other hand, C18:0 concentration was decreased proportionally to the addition of OMW and ^Δ9^C16:1 concentration value presented a noticeable fall in the blended cultures with Ph_o_~3.5 g/L (compared to the rest of the experiments) (Table 2). Finally, to estimate the fatty acid desaturase activity during yeast cultivation, the ratios between the desaturase product and substrate (C16:1/C16:0, C18:1/C18:0, C18:2/C18:1) were calculated (data not shown). High values of the C18:1/C18:0 ratio indicate high activity of Δ9-desaturase, especially in the citric acid production phase. In all cultures (control and blended) those ratios (mainly C18:1/C18:0) were raised at the citric acid production phase (~144 h) compared to yeast growth phase (72 h).

#### 2.1.4. Decolorization—Removal of Phenolic Compounds

**Figure 2:**
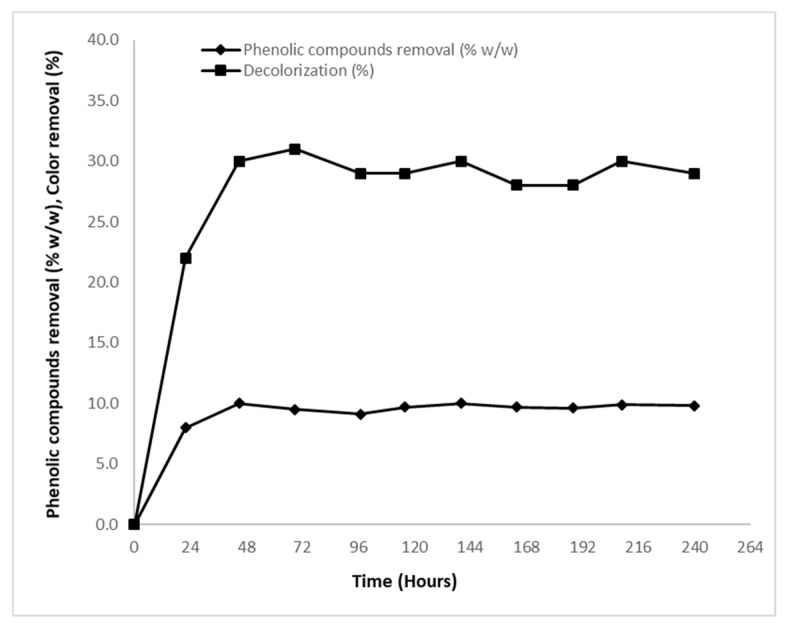
Phenolic compounds removal (% *w*/*w*) (-♦-) and color removal (%) (-■-), during growth of *Yarrowia lipolytica* strain ACA-DC 5029 on crude glycerol with initial concentration of ~70 g/L, blended with olive mill wastewaters offering initial phenolic compounds concentration of ~3.5 g/L. Culture conditions as described in Table 1. Each point is the mean value of two independent measurements.

Even though natural yeast strains do not possess the ability to produce phenol-oxidizing enzymes [12,16], partial decolorization of blended media (dark color due to OMW presence) and slight removal of phenolic compounds occurred in the present study. Specifically, in all cases, *Y. lipolytica* strain ACA-DC 5029 presented the ability to remove phenolic compounds up to ~10% *w*/*w* (~0.4 g/L maximum removal of phenols in culture with Ph_0_~3.5 g/L) and color up to ~30%, compared to media composition prior to fermentation (Figure 2). This is a crucial result as it has been reported that the limiting step of OMW remediation is the breakdown of its phenolic compounds (and therefore the removal of OMW color) [1,3,4,12]. This fact could be attributed to potential adsorption of phenolic compounds in the yeast cells (through weak and reversible interactions, mainly between anthocyanins and yeast walls) [48] or even to partial utilization of phenolic compounds as carbon and energy source [49].

### 2.2. Growth of Y. lipolytica Strain ACA-DC 5029 in Media with Initial Crude Glycerol Concentration of ~120 g/L and of ~170 g/L.

#### 2.2.1. Biomass and Intra-Cellular Polysaccharides (IPS) Evolution

In trials with initial glycerol concentration of ~70 g/L, as mentioned above, the strain presented satisfactory growth with maximum biomass value of 11.96 g/L (Y_X/Glol_ = 0.17 g/g). With a view to valorize larger volumes of crude glycerol and potentially enhance the production of cellular metabolites (i.e., citric acid and/or cellular lipid and/or polyol production), cultures with high excess of carbon (with nitrogen concentration remaining stable) were conducted. Thus, trials were performed with increased initial crude glycerol concentration to ~120 g/L (total substrate assimilation in 360 h; 15 days) and to ~170 g/L (total substrate assimilation in 528 h; 22 days). In both experiments, crude glycerol was completely assimilated. As reported in Table 3, maximum biomass production (X*max*) reached 12.40 g/L with respective yield of biomass per glycerol consumed (Y_X/Glol_) 0.10 g/g in cultures with Glol_0_~120 g/L and 12.48 g/L (Y_X/Glol_ = 0.08 g/g) in cultures with Glol_0_~170 g/L. Carbon excess conditions seemed not to affect biomass production in terms of absolute values. Nevertheless, the yield of biomass per glycerol consumed (Y_X/Glol_) was significantly reduced (see Table 3). 

**Figure 3:**
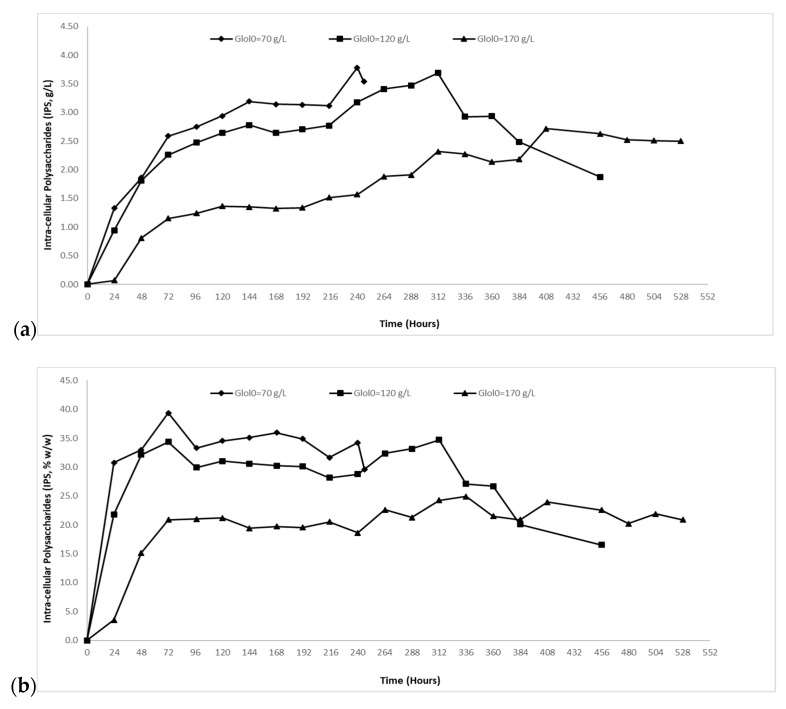
(**a**) Intra-cellular polysaccharides (IPS, g/L) and (**b**) yield of intra-cellular polysaccharides produced on biomass produced (% *w*/*w*) evolution during growth of *Yarrowia lipolytica* strain ACA-DC 5029 on media with ~70 g/L (-♦-), ~120 g/L (-■-) and ~170 g/L (-▲-) initial crude glycerol concertation. Culture conditions as described in Table 1. Each point is the mean value of two independent measurements.

At this set of trials, the excess of carbon in the media seemed not to affect IPS production, except in the trial with the highest initial crude glycerol concentration (Glol_0_~170 g/L) (Figure 3a,b). Specifically, IPS_max_ concentration was 3.78 g/L with subsequent yield in dry weight 34.2% *w*/*w* in the experiment with Glol_0_~70 g/L. In cultures with Glol_0_~120 g/L, IPS_max_ value was 3.69 g/L and yield in dry weight 34.7% *w*/*w* (the highest achieved in this study) (Figure 3a,b). In cultures with Glol_0_~170 g/L IPS_max_ value was 2.72 g/L and yield in dry weight 23.9% *w*/*w*. In cultures with Glol_0_~70 g/L and ~120 g/L endo-polysaccharide formation was gradually increased up to ~30.0% *w*/*w* until 48 h after inoculation. In the case of cultures with Glol_0_~70 g/L the yield seemed to remain constant at ~30.0–34.0% *w*/*w* until the end of fermentation (Figure 3b). On the other hand, in the case of cultures with Glol_0_~120 g/L the yield seemed to remain constant at ~30.0–34.0% *w*/*w* until 312 after inoculation, after which it started to gradually decrease until the end of fermentation (Figure 3b). In Glol_0_~170 g/L, IPS accumulation increased gradually up to ~20% *w*/*w* until 72 h after inoculation and remained constant at ~20.0–24.0% *w*/*w* (Figure 3b).

#### 2.2.2. Citric Acid and Polyols Production

**Figure 4:**
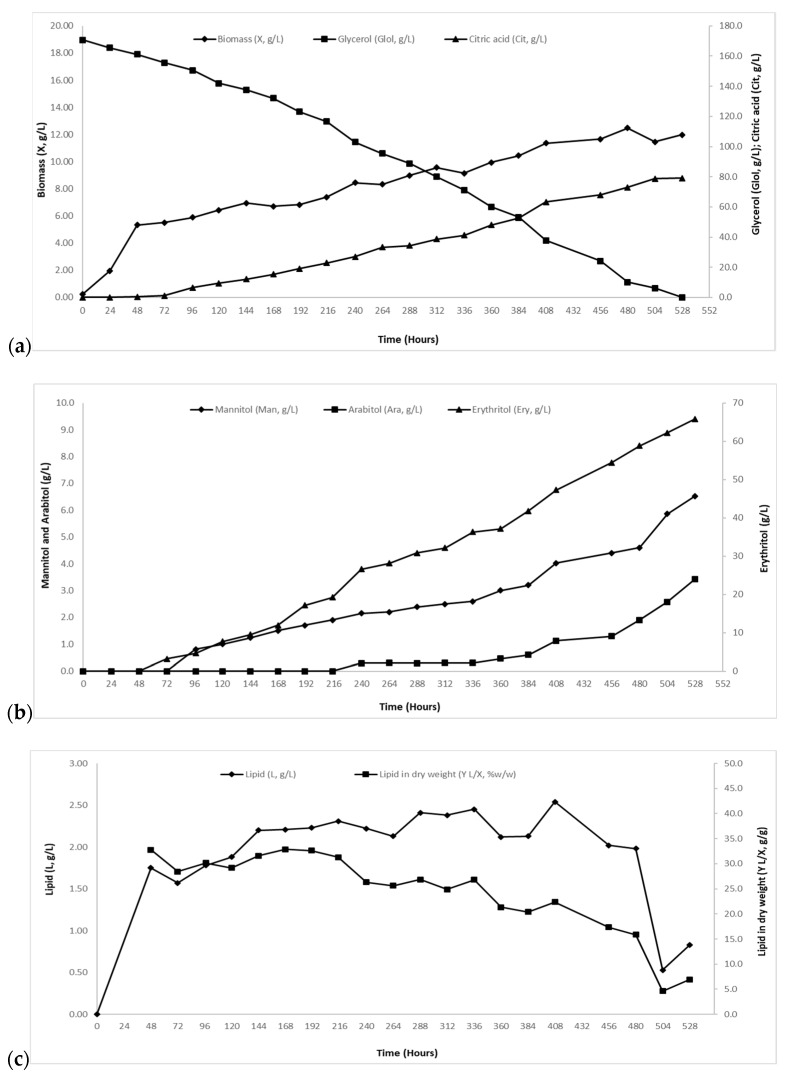
(**a**) Biomass (X, g/L) (-♦-), glycerol (Glol, g/L) (-■-) and citric acid (Cit, g/L) (-▲-); (**b**) mannitol (Man, g/L) (-♦-), arabitol (Ara, g/L) (-■-) and erythritol (Ery, g/L) (-▲-); (**c**) cellular lipids (L, g/L) (-♦-) and cellular lipid in dry weight (Y_L/X_, % *w*/*w*) (-■-) evolution during growth of *Yarrowia lipolytica* strain ACA-DC 5029 on media with ~170 g/L initial crude glycerol concertation. Culture conditions as described in Table 1. Each point is the mean value of two independent measurements.

As mentioned above, in experiments with Glol_0_~70 g/L, citric acid secretion reached the maximum value (Cit_max_) of 42.5 g/L with respective yield of citric acid per glycerol consumed (Y_Cit/Glol_) 0.59 g/g (Table 3). In crude glycerol excess media, a significant raise in citric acid accumulation was noted reaching Cit_max_ = 63.8 g/L (Y_Cit/Glol_ = 0.52 g/g; cultures with Glol_0_~120 g/L; Table 3) and Cit_max_ = 79.0 g/L (Y_Cit/Glol_ = 0.46 g/g; cultures with Glol_0_~170 g/L; Table 3 and Figure 4a); these values were the highest ones in this study. Nevertheless, the conversion yield of citric acid produced per glycerol consumed was clearly decreased in comparison with trials with the lowest initial crude glycerol concentration (~70 g/L). 

Erythritol secretion was highly favored by the increase of glycerol concentration in the media reaching 65.8 g/L (the highest reported value in this study) and Y_Ery/Glol_ = 0.39 g/g (see Table 3 and Figure 4b) in the experiment with Glol_0_~170 g/L. In terms of the cultures with Glol_0_~120 g/L, Ery_max_ concentration reached the value of 38.4 g/L (Y_Ery/Glol_ = 0.39 g/g) and in cultures with the lowest initial crude glycerol concentration (Glol_0_~70 g/L), Ery_max_ concentration reached 14.9 g/L (Y_Ery/Glol_ = 0.23 g/g) (Table 3). These observations indicated a shift of the yeast metabolism from citric acid accumulation towards erythritol production. In the literature, it has been reported that polyols function as osmolytes stored inside microbial cells (by means of cell protection by osmotic stress), thus act as soluble compounds that maintain the cell’s fluid balance and protein folding [23,26,46,50]. Likewise, in the present study, the substantial enhancement of erythritol production (Table 3), might be attributed to the exposure of the selected strain into higher glycerol concentrations.

Arabitol secretion was limited (Table 3; see also Figure 4b), since the increased glycerol concentrations did not significantly affect its production. Maximum concentration values were in the range of 3.2–4.5 g/L (Y_Ara/Glol_ = 0.02–0.04 g/g) (Table 3). On the other hand, mannitol production was reduced proportionally to the excess of carbon in the media. Overall, Man_max_ concentration reached the value of 10.1g/L (Y_Man/Glol_ = 0.14 g/g) in fermentations with the lowest Glol_0_ (~70 g/L), whereas in the cultures with Glol_0_~120 g/L, Man_max_ was 8.0 g/L (Y_Man/Glol_ = 0.08 g/g) and in the cultures with Glol_0_~170 g/L Man_max_ was 6.5 g/L (Y_Man/Glol_ = 0.04 g/g) [Figure 4b].

#### 2.2.3. Biogenesis of Cellular Lipids and Fatty Acid (FA) Composition

In the experiments with increased crude glycerol concentrations, maximum cellular lipid concentration (L_max_) reached 1.27–2.54 g/L with total lipid in dry biomass (Y_L/X_) values of 10.6–22.4% *w*/*w* (Table 3). In antithesis with fermentations with low Glol_0_ concentration (~70 g/L), the addition of excess crude glycerol into the media seemed to shift the cell metabolism from citric acid accumulation not only toward erythritol secretion (see Section 2.2.2.) but also toward lipid production. For instance, lipid production was enhanced reaching 2.54 g/L and respective yield lipid in biomass of 22.4% *w*/*w* in cultures with Glol_0_~170 g/L, whereas L_max_ reached 2.07 g/L and respective yield lipid in biomass of 19.7% *w*/*w* in cultures with Glol_0_~120 g/L (Table 3). The accumulation of citric acid and cellular lipids from various carbon sources is a non-growth associated process, which occurs after the exhaustion of nitrogen source from the medium [23,46]. This means that cellular metabolism is directed towards the biosynthesis of citric acid and lipids proportionally to the carbon excess in the substrate [51,52,53,54,55]. In the present study, cell metabolism was directed towards the biosynthesis of erythritol (instead of citric acid) and microbial oil. It is worth noticing the fact that that in cultures with Glol_0_~170 g/L, reserve lipid was vastly re-consumed ~504 h after inoculation [Figure 4c], which was also the time point where glycerol was depleted from the culture medium. Microbial lipid concentration dropped to ~0.53 g/L (Y_L/X_~4.6% *w*/*w*) from ~2.54 g/L (Y_L/X_~22.4% *w*/*w*) possibly in favor of additional polyol production [Figure 4b,c].

The fatty acid (FA) composition of intra-cellular lipids was analyzed at various growth phases for all cultures (Table 4). As expected, the principal FAs detected were the C16 and C18 ones. In all cultures UI of FA changed within culture phases and seemed to increase proportionally to the evolution of the culture as the concentration of ^Δ9^C16:1, ^Δ9^C18:1 and ^Δ9,12^C18:2 increased and the concentration of C16:0, C18:0 decreased (or remained stable). The addition of higher volumes of crude glycerol in the media seemed to increase the UI (Table 3). Moreover, this addition led to an increase of the concentration of ^Δ9^C18:1 (compared to cultures with Glol_0_~70.0 g/L). In the cultures with Glol_0_~120.0 g/L the concentration of oleic acid reached the value of ~63% *w*/*w* and in the cultures with Glol_0_~170.0 g/L ^Δ9^C18:1 concentration was ~53% *w*/*w*. Likewise, palmitoleic acid concentration increased proportionally to crude glycerol addition (Table 4). It should be noted that, compared to cultures with Glol_0_~70.0 g/L, the excess of carbon source into the media (Glol_0_~120.0 g/L) favored the synthesis of ^Δ9,12^C18:2, as it raised from ~14.0% *w*/*w* in the former case up to ~52.0% *w*/*w* in the latter. Surprisingly, the concentration of linoleic acid was reduced in cultures with Glol_0_~170.0 g/L (Table 4). As stated previously in this study, to estimate the fatty acid desaturase activity during yeast cultivation, C16:1/C16:0, C18:1/C18:0, C18:2/C18:1 ratios were calculated (data not shown). High values of C18:1/C18:0 ratio were noted, indicating a stimulated activity of Δ9-desaturase, especially in the citric acid production phase. In all cultures those ratios were raised at the citric acid production phase compared to yeast growth phase. Increased Glol_0_ into the media led to a clear increase of all ratios (except C18:2/C18:1 ratio in the trial with Glol_0_~170 g/L) leading to the conclusion that (Δ9- and Δ12-) desaturase activity was stimulated proportionally to crude glycerol addition in the cultures.

## 3. Discussion

*Y. lipolytica* strain ACA-DC 5029 was evaluated for its ability to grow on nitrogen-limited submerged shake-flasks cultures containing blends of OMW (a food industry waste stream) and crude biodiesel derived glycerol (constant Glol_0_~70 g/L and various volumes of OMW used, offering Ph_0_~1.0 g/L, 2.0 g/L and 3.5 g/L), in order to produce citric acid, cellular lipid and polyols. At the same time, color removal and phenolic compound concentration reduction in the media was monitored. To the best of the authors’ knowledge, this is the first report in literature in which blends of OMW and crude biodiesel-derived glycerol are used as substrate for (high-) added value compounds by yeasts; blends of OMW and pure glycerol have been previously reported [33]. Additionally, experiments including media with higher Glol_0_ concentration of ~120 g/L and of ~170 g/L were conducted. The overall aim of the study was to investigate the impact of carbon excess culture conditions on the kinetic behavior of the strain and to assess the potential of valorizing greater volumes of OMW, by means of subsequent enhancement of microbial metabolites production.

*Y. lipolytica* strain ACA-DC 5029 presented satisfactory growth in OMW and crude glycerol blends. Increased phenolics concentrations (due to larger volumes of OMW added) did not affect biomass production (X_max_~11.6 g/L, Y_X/Glol_~0.17–0.18 g/g) compared to the control experiment (X_max_ = 11.96 g/L, Y_X/Glol_~0.17 g/g) except for the trial with the highest Ph_0_~3.5 g/L (X_max_ = 8.72 g/L, Y_X/Glol_~0.13 g/g). In line with the results of the present study, no significant differences in biomass production were noted, when *Y. lipolytica* strain ACA-DC 50109 was grown on OMW-based nitrogen-limited media (enriched with commercial glucose) with Ph_0_~0.7–1.8 g/L [16], when *S. cerevisiae* strain MAK 1 was grown on blends of OMW and molasses with Ph_0_~2.6–6.3 g/L [19] and when selected Zygomycetes strains (*Mortierella isabellina* MUCL 15102, *M. ramanniana* MUCL 9235, *Cunninghamella echinulata* ATHUM 4411, *Mucor* sp. LGAM 365, *Thamnidium elegans* CCF-1465 and *Zygorhynchus moelleri* MUCL 1430) were grown on solidified media with OMW up to 50% (*v*/*v*) [8]. In contrast, in a study where *Y. lipolytica* strain ACA-YC 5033 was grown on OMW-based nitrogen-limited media (enriched with commercial glucose) with Ph_0_~1.8–2.0 g/L, biomass values were depleted in parallel with increased phenolic content of the medium [34]. Moreover, when OMW-based nitrogen-limited media (enriched with commercial glucose) with Ph_0_~1.0–1.6 g/L were used for the growth of three *Y. lipolytica* strains, two of them (*Y. lipolytica* strain ACA-YC 5028 and ACA-YC 5033) were negatively affected by OMW addition, whereas the third one (*Y. lipolytica* strain W29) presented high biomass values with the increment of OMW [13]. It could be assumed that the yeast tolerance against the presence of phenolic compounds into the media, is strain-dependent. 

The microbial production of citric acid on OMW-based media has been scarcely reported [13,16,33,34]. There are studies (considering use of OMW-based media) where citric acid production by some strains remained unaffected (up to a moderate Ph_o_ concentration) [13,34] whereas in others is reduced by the addition of OMW [13]. In the present study, it is assumed that the addition of OMW in the media affected negatively citric acid production. Nevertheless, in blended media, it seems that OMW addition favored the accumulation of citric acid (Table 1). Such a fact may be of great importance since in a potential process scale-up OMW could partially or in some cases totally substitute tap water for glycerol dilution, thus offering an attractive alternative in terms of preserving water resources. When comparing the values of citric acid production of this study (when OMW and crude glycerol blends used as substrate) with literature (where glycerol was used as carbon source; see below) the achieved values are regarded as low. However, if citric acid production is compared with the few studies that include OMW-based media (to produce extracellular metabolites and specifically citric acid), the values of the present study are among the highest reported. Specifically, in the present study, Cit_max_ value was 37.4 g/L (Y_Cit/Glol_ = 0.55 g/g) in culture with initial phenolic compound concentration of ~3.5 g/L. In Dourou et al. [33], when *Y. lipolytica* strain LGAM S (7) was grown on OMW and pure glycerol blends the Cit_max_ value achieved was 30.3 g/L (Y_Cit/Glol_ = 0.62 g/g). When *Y. lipolytica* strain ACA-DC 50109 used OMW-based media enriched with commercial glucose, Cit_max_ value achieved was 28.9 g/L (Y_Cit/Glol_ = 0.53 g/g). Likewise, when *Y. lipolytica* strain W29 [13] and *Y. lipolytica* strain ACA-YC 5033 grown OMW-based media enriched with commercial glucose, Cit_max_ values achieved were 15.8 g/L (Y_Cit/Glol_ = 0.46 g/g) and 52.0 g/L (Y_Cit/Glol_ = 0.64 g/g) respectively. 

The strain *Y. lipolytica* ACA-DC 5029, presented the ability to produce the polyols mannitol, arabitol and erythritol when crude glycerol was used as substrate, in both control and blended cultures (regarding OMW and glycerol blends: Man_max_ = 13.1 g/L; Y_Man/Glol_ = 0.21 g/g; Ara_max_ = 3.1 g/L; Y_Ara/Glol_ = 0.05 g/g; Ery_max_ = 13.5 g/L; Y_Ery/Glol_ = 0.19 g/g). Mannitol and arabitol production was negligibly affected by the addition of OMW into the media, compared with control trials. On the other hand, erythritol production was vastly reduced when OMW was added into the media (Ery_max_ = 2.4 g/L, Y_Ery/Glol_ = 0.04 g/g; Ph_0_~3.5 g/L), compared to control cultures (Ery_max_ = 14.9 g/L, Y_Ery/Glol_ = 0.23 g/g). There seems to be a shift of the metabolism towards citric acid production at the expense of mainly erythritol production, due to the addition of OMW into the medium [though exception was the trial with initial phenolic compound concentration of ~2.0 g/L, where the value of Ery_max_ was 13.5 (Y_Ery/Glol_ = 0.19 g/g)]. This is a result that cannot be further explained since the present study is the first report in literature including blended media of crude glycerol and OMW (besides the study of Dourou et al. [33] where blends of OMW and pure glycerol were used) dealing specifically with erythritol accumulation.

The yeast strain used in this study presented a shift of its metabolism towards lipid accumulation since a notable increase in cellular lipid production proportional to the addition of volumes of OMW into the blended media occurred. Increasing quantities of intra-cellular lipids were secreted (L_max_ = 1.32–1.95 g/L, Y_L/X_~16.0–20.0% *w*/*w*) compared to control experiments (L_max_ = 1.27 g/L, Y_L/X_~11.0% *w*/*w*). These findings are in full accordance with literature regarding other *Y. lipolytica* strains growing on OMW-based media where the presence of OMW favored notably storage lipid production process [13,34]. Apart from *Y. lipolytica* strains, microorganisms belonging to other oleaginous genera (e.g., Zygomycetes) have presented similar physiological behavior [8]. Therefore, OMW could be considered as a “lipogenic” medium. It should be noted also that a scarce number of studies indicate the significant enhancement of lipid accumulation process in yeast cells by the addition of natural compounds (e.g., *Teucrium polium* L. aqueous extracts, Origanum extracts, etc) [56,57]. Evidently, this kind of studies present academic and economic interest. 

Partial decolorization of blended media (dark color due to OMW presence) and slight removal of phenolic compounds occurred. In all trials, *Y. lipolytica* strain ACA-DC 5029 presented the ability to remove phenolic compounds up to ~10% *w*/*w* and color up to ~30% *w*/*w*, compared to the media composition prior to fermentation. The reduction of OMW color and removal of their phenolic content by yeasts appear to be a strain-dependent process; however, the exact mechanism through which this is achieved is not yet clear. Some strains belonging to *Saccharomyces* and *Yarrowia* genera possess the ability to significantly reduce color, and to lesser extent remove phenolic compounds from the media [1,13,16,19,20,34,58]. Other strains seem incapable, when grown on OMW-media to reduce phenolics concentration and color [15,33,59]. The bioremediation of OMW- based media by various yeast strains is summarized elsewhere [34]. Literature reports the existence of microorganisms presenting the ability to reduce phenolic content of OMW-based media such as *Geotrichum candidum* [60,61,62], *Pleurotus ostreatus* [4], *Aspergillus niger* [12,62], *Candida boidinii* [60], *Yarrowia lipolytica* [13,16,33,34] and *Saccharomyces cerevisiae* [19,20]. Higher fungi present the ability to oxidize phenolic compounds due to the fact that extra-cellular oxidases (ligninolytic enzymes) laccases, lignin peroxidases and manganese dependent (or independent) peroxidases are being secreted [4,12,63]. On the other hand, yeasts lack the ability of producing such enzymes to break down phenolic compounds [4,63,64,65]. Production of laccases has been achieved only in genetically engineered *Y. lipolytica* strains [66]. Nevertheless, significant reduction of phenolic compounds in cultures performed by yeast species, is scarcely reported [34,49,58,67]. Therefore, both the reduction of phenolic compounds and removal of color of OMW should not be attributed to phenol-oxidizing mechanisms, since yeasts do not inherently possess such enzymes [4]. This fact could be attributed to potential adsorption of phenolic compounds in the yeast cells (by establishment of weak and reversible interactions, mainly between anthocyanins and yeast walls) [48] or even to partial utilization of phenolic compounds as carbon and energy source [49].

In experiments with increased crude glycerol concentration substrate was totally assimilated in 15 and 22 days respectively. In cultures with Glol_0_~70 g/L (X_max_~12.00 g/L, Y_X/Glol_~0.17 g/g; L_max_~1.30 g/L, Y_L/X_~11.0% *w*/*w*) biomass production was not affected but the yield of biomass on glycerol consumed was significantly reduced (Y_X/Glol_ = 0.08–0.10 g/g) (Table 3). On the other hand, microbial lipid accumulation was enhanced (L_max_~2.50 g/L, Y_L/X_~22.0% *w*/*w* in cultures with Glol_0_~170 g/L; L_max_~2.10 g/L, Y_L/X_~20.0% *w*/*w* in the culture with Glol_0_~120 g/L). As microbial lipid and citric acid accumulation is considered as a non-growth associated process which occurs after the exhaustion of nitrogen source from media [23,46], such results could be expected. Microbial metabolism is directed towards the biosynthesis of citric acid and lipids proportionally to the excess of carbon substrate [51,52,53,54,55]. Natural or genetically engineered *Y. lipolytica* strains grown on hydrophilic media (such as glycerol, glucose etc.) under nitrogen-limited conditions are reported to present microbial lipid break-down even if a carbon source still in significant concentrations in the media. Simultaneously, quantities of low-molecular weight compounds (mostly citric acid but also polyols) were accumulated [23,68,69,70]. Likewise, in this study in the cultures with Glol_0_~170 g/L, when glycerol was depleted from the culture, reserve lipid was vastly (~80%) re-consumed ~504 h after fermentation has started (Figure 4c) while citric acid and polyols continued to be produced (see Figure 4). Microbial lipid concentration dropped to ~0.50 g/L (Y_L/X_~5.0% *w*/*w*) from ~2.50 g/L (Y_L/X_~22.0% *w*/*w*). Similarly, in a study where *Y. lipolytica* strains cultivated in crude glycerol, microbial oil was re-consumed towards the production of citric acid (*Y. lipolytica* strain ACA-YC 5033) and blends of citric acid and mannitol (*Y. lipolytica* strain ACA-YC 5029) [71]. In another report [70], when *Y. lipolytica* strain ACA-YC 5030 was grown on biodiesel-derived glycerol, the yield of lipid in biomass presented elevated values (until their depletion as the fermentation proceeded) while mannitol and erythritol were secreted into the medium.

Citric acid production was raised proportionally to the addition of crude glycerol in the media, as expected. The highest value of Cit_max_ achieved in this study was 79.0 g/L (Y_Cit/Glol_ = 0.46 g/g) in cultures with Glol_0_~170 g/L. On the other hand, values of Y_Cit/Glol_ were critically reduced with the excess of carbon in the media suggesting a shift of yeast metabolism from citric acid accumulation towards erythritol production. Comparing with other related studies (where glycerol was used as carbon source) presented in international literature where maximum citric acid values reported are in the range of 19–154 g/L with respective conversion yields of citric acid produced per glycerol consumed being 0.44–0.92 g/g [28,33,51,69,71,72,73,74,75,76,77,78], it could be assumed that citric acid production in this study can be classified as relatively satisfactory (for data regarding citric acid production in various fermentation types, when *Yarrowia lipolytica* strains grown on glycerol-based media see Papanikolaou et al. [69]). Erythritol synthesis was highly favored reaching the maximum value of 65.8 g/L (Y_Ery/Glol_ = 0.39 g/g) in the experiment with Glol_0_~170 g/L whereas in cultures with the lowest initial crude glycerol concentration (Glol_0_~70 g/L), Ery_max_ concentration reached the value of 14.9 g/L (Y_Ery/Glol_ = 0.23 g/g). When cells encounter osmotic stress, sugar alcohols biosynthesis might protect them. Polyols might have the ability to function as osmolytes (stored inside cells), thus act as soluble compounds maintaining the cell’s fluid balance and correct protein folding [23,26,46,50]. Such fact might be attributed to the exposure of the strain into relatively high glycerol concentrations of the media used in the present study. Another issue that should be stressed out is that in contrast with other studies [77,79,80], despite the fact that media pH remains over 5.0, erythritol production is increased proportionally to glycerol addition into the media. In Chatzifragkou et al. [81], mannitol synthesis was favored when higher concentrations of glycerol were applied, while pH was maintained in the range of 5.0–6.0. In the trial with Glol_0_~70 g/L, after exhaustion of glycerol, erythritol (and to a lesser extent mannitol) was partially re-consumed in favor of citric acid production (Table 1). In another study, when *Y. lipolytica* strain LFMB 20 cultivated on biodiesel-derived glycerol (Glol_0_~100 g/L) erythritol re-consumption was observed while a quantity of the substrate (~34 g/L) remained unconsumed. While, glycerol continued to be assimilated, mannitol constant synthesis maintained and citric acid synthesis in elevated quantities occurred [82]. In Chatzifragkou et al. [81], partial re-consumption of mannitol (and other metabolites) by *Y. lipolytica* strain LFMB 19 when grown in crude glycerol was noted. Biomass concentration did not raise any further, suggesting that this degradation was performed in favor of energy of maintenance requirements. The biosynthesis of polyols using glycerol as substrate is not completely clear [40,41], while hypothetical pathways of glycerol conversion into polyhydroxy alcohols in *Y. lipolytica* are suggested by Tomaszewska et al. [40]. Research on biosynthesis of polyols by yeasts strains cultivated on glycerol (especially crude) are on-going in the last decade, but nevertheless limited. Some studies report the production of mannitol, arabitol and erythritol by *Y. lipolytica* strains when cultivated on crude glycerol in various modes. The maximum values of arabitol concentration achieved were ~0.3–6.0 g/L (versus 0.3–5.6 g/L in present study), of mannitol ~1.0–41.0 g/L (versus 5.3–13.1 g/L in present study) and of erythritol ~13.0–170.0 g/L [23,38,40,41,57,70,71,76,79,80,81,82,83,84]. Compared to present study, maximum erythritol production (Ery_max_=65.8 g/L in the culture with Glol_0_~170 g/L) should be considered as relatively satisfactory. Especially when compared with batch bioreactor cultures (Ery_max_~58.0–81.0 g/L) [40,41,84] the erythritol concentration value achieved in this study is one of the highest reported. When compared to results obtained in shake-flasks cultures (Ery_max_~13.0–36.0 g/L) [70,82], the maximum erythritol concentration of this study is the highest reported so far.

Biosynthesis of citric acid and/or SCO from glycerol (or other similarly metabolized compounds such as glucose) is triggered when nitrogen is depleted from the culture medium [29,85] and is biochemically equivalent in the first steps [69]. When carbon is excess and nitrogen is limited, oleaginous microorganisms produce large amounts of TCA cycle intermediates, including citric acid and iso-citric acid, which are not further catabolized. The biochemical mechanism that describes this phenomenon is presented elsewhere (for reviews see: Papanikolaou and Aggelis [86,87], Beopoulos et al. [88]). This biosynthesis seems to be strain-dependent as in some cases (in flask or in bioreactor experiments), SCO is produced in high yields (Y_L/X_ > 35% *w*/*w*) [89,90,91] whereas in other reports relatively low quantities of lipids are synthesized (Y_L/X_ < 22% *w*/*w*) but metabolites such as citric acid and other low-molecular metabolites (i.e., polyols) are secreted into the growth medium [26,51,68,78,80,92]. In some cases when batch cultures of *Y. lipolytica* were performed under carbon-excess conditions (de novo lipid accumulation), a biosynthesis “interplay” between lipids and citric acid occurred. Specifically, even though nitrogen was present in the first fermentation steps, some accumulation of lipid was observed. Despite the significant presence of substrate into the medium, lipid degradation (turnover) occurred simultaneously with the secretion of citric acid and polyols [51,68,69,70,81,82,92]. In some other cases, citric acid and lipids where simultaneously produced [23,93].

With regards to the composition of microbial lipid produced by the yeast strain, the main FAs of lipids accumulated, on blended media (OMW and glycerol mixtures) and in control media (no OMW but only glycerol added), were C16 and C18 aliphatic chains. Palmitic (C16:0), palmitoleic (^Δ9^C16:1), oleic (^Δ9^C18:1) and linoleic (^Δ9,12^C18:2) acids were the predominant FAs. This is in accordance with some cases in international literature where *Y. lipolytica* strains were grown on OMW-based media [13], on biodiesel derived waste glycerol [73,94] and on blends of OMW and pure glycerol [33]. Nevertheless, when *Y. lipolytica* strains use hydrophilic compounds (glucose, glycerol, ethanol etc.) as substrate, the distribution of intra-cellular FAs seems to be generally strain-dependent. There are also other parameters influencing the FA composition of lipids produced by *Y. lipolytica* strains, such as the initial (for batch cultures) or the inlet (for continuous cultures) substrate concentration, the fermentation time and the dilution rate [23,51,56,68,95]. Regarding growth of *Y. lipolytica* strains on OMW-based media, it should be noted that similarly with the present study, concentration of oleic acid increased (together with palmitoleic acid linoleic acid) with the addition of OMW into the media [13,16,33,34]. Rather than yeast *Y. lipolytica*, this feature seems also to happen with other yeast species (such as *S. cerevisiae*) [19]. These findings could be attributed to microbial adaptation on the high-phenol concentration media.

## 4. Materials and Methods

### 4.1. Microorganism and Growth Media

In the present study, *Yarrowia lipolytica* strain ACA-DC 5029 belonging to the collection of Laboratory of Dairy Research Department of Food Science and Human Nutrition, Agricultural University of Athens, was used. The strain was grown on media including blends of OMW and crude industrial glycerol (deriving as by-product from biodiesel production) and tested towards its ability to produce biomass, citric acid, polyols and lipids. It was conserved in yeast peptone dextrose agar (YPDA; T = 6 ± 1 °C) and for viability maintenance, it was sub-cultured every 2 months. OMW was received from a three-phase decanter olive mill located in Chania (Crete, Greece) and were frozen at T = −20 ± 2 °C. Prior experimental use, OMW was de-frozen and the solids removed by centrifugation (9000 rpm, 15 min, T = 21 ± 1 °C) in a Universal 320R centrifuge (Hettich, Tuttlingen, Germany). OMW phenolic content expressed as gallic acid equivalent was ~3.5 g/L, while (surprisingly enough) no reducing sugars were found (therefore crude glycerol was the sole carbon source in the blended media). Moreover, negligible quantities of olive oil (~0.2 g/L—determination of oil conducted after triple extraction with hexane) were present. Crude glycerol with a purity of ~81% *w*/*w* was provided by Agroinvest S.A. (Achladi, Fthiotida Prefecture, Greece). The impurities were composed of: 11–12% *w*/*w* water, 5–6% *w*/*w* potassium salts, 1% *w*/*w* free-fatty acids and less than 0.2% *w*/*w* methanol. In the trials regarding blended media (OMW and crude glycerol added), the initial glycerol (Glol_0_) concentration was set to ~70 g/L. OMW was used as process tap water substitute to dilute glycerol. Thus, specific volumes of OMW and tap water were added in the blended media, offering initial concentration of phenolic compounds (Ph_0_) of ~1.0 g/L, ~2.0 g/L and ~3.5 g/L (in the latter case, no tap water was added but only OMW). A control experiment (no OMW addition) was also performed. To investigate the impact of higher glycerol concentration and increased carbon excess on kinetics of the strain, shake-flasks control experiments (no OMW addition) with media containing Glol_0_ concentrations (in g/L): ~120 and ~170 g/L, were conducted. All other culture parameters remained stable. Mineral salts were added into the media (concentration in g/L): KH_2_PO_4_ 7.00; Na_2_HPO_4_ 2.50; MgSO_4_·7H_2_O 1.50; FeCl_3_·6H_2_O 0.15; CaCl_2_·2H_2_O 0.15; ZnSO_4_·7H_2_O 0.02; MnSO_4_·H_2_O 0.06. As nitrogen source, peptone and yeast extract were used in a concentration of 1.0 g/L each, imposing nitrogen-limited conditions in all trials.

### 4.2. Culture Conditions

All cultures performed in 250 mL Erlenmeyer flasks containing 50 ± 1 mL of sterilized growth medium. Media were inoculated with 1 mL (2% *v*/*v* inoculum) of exponential (24 h) pre-culture. The pre-culture was carried out in a synthetic medium containing glucose ~10 g/L, peptone ~10 g/L and yeast extract ~10 g/L. All fermentations carried out under fully aerobic conditions [Dissolved Oxygen Tension (DOT) > 20% *v*/*v*] as flasks were placed in an orbital shaker (New Brunswick Scientific Co., Edison, NJ, USA) at an agitation rate of 180 ± 5 rpm (T = 28 ± 1 °C). Flasks were incubated aerobically in an orbital shaker at an agitation rate of 180 ± 5 rpm and T = 28 ± 1 °C, thus, all cultures were performed under fully aerobic conditions [Dissolved Oxygen Tension (DOT) > 20% *v*/*v*]. It should be noted that pH of all cultures has been standardized at the value 6.0, prior to inoculation. As described in Sarris et al. [13], by adding measured quantities of 5M NaOH, pH of the culture maintained between the values of 5.0 and 6.0. All trials performed in duplicate (each experimental point of the kinetics presented is the mean value of two independent determinations; SE < 10%).

### 4.3. Analytical Methods

Yeast cells were harvested from broth by centrifugation [9000 rpm, 10 min, 4 °C; (Universal 320R Centrifuge, Hettich, Tuttlingen, Germany)], washed once with distilled water and centrifuged again. The determination of biomass concentration was from dry weight (~85 °C until constant weight). Cellular lipid was determined gravimetrically using a mixture of chloroform/methanol 2/1 (*v*/*v*) as the extracting solvent, as described in Sarris et al. [13]. Subsequently, lipids were converted into their fatty acid methyl-esters (FAMEs) and qualitative analysis took place in a gas chromatograph (8060 unit, Fisons Instruments Inc., Danvers, MA, USA) with flame ionization detector (FID) and using a Chrompack column (60 m × 0.25 mm, film thickness 0.25 mm, J&W Scientific Inc., Folsom, CA, USA), according to Fakas et al. [96]. FAMEs were identified by comparison with authentic standards. DOT was determined off-line with the aid of a selective electrode (oxi200 Sensodirect, Lovibond, Amesbury, UK) according to a protocol suggested by Papanikolaou et al. [97]. The determination of total intra-cellular polysaccharides (IPS) were conducted as described in Tsakona et al. [98] but slightly modified. Briefly, 0.05 g of dry yeast biomass was initially disrupted by adding 10 mL of 2M HCl. Heat treatment (100 °C) followed for 30 min. Cellular debris was removed by filtration and the neutralization of the filtered liquid took place by adding 10 mL of 2 M NaOH up to pH 7.0. The concentration of total sugars was determined with the assay of 3,5-dinitrosalicylic acid [99] and IPS were expressed as glucose equivalents. Filtered aliquots of the supernatant culture medium including glycerol, citric acid, mannitol, arabitol and erythritol were analyzed with the aid of a Waters 600E HPLC system (Waters Association, Milford, MA, USA) using a 30.0 cm × 7.8 mm Rezex ROA column (Phenomenex, Torrance, CA, USA) coupled to a differential refractometer (RI; Waters 410) and a UV detector (Waters 486) (sample volume 20 μL; mobile phase 5 mM H_2_SO_4_; flow rate 0.6 mL/min; column temperature T = 65 °C. Phenolic compounds concentration in the fermentation supernatant was determined according to the Folin–Ciocalteau method measured at 750 nm and expressed as gallic acid equivalent [4]. The decolorization assay of the treated media was performed according to Sayadi and Ellouz [6]. The samples were diluted 30-fold, the pH adjusted between 6.0 and 6.3 and the absorbance measured at 395 nm. In all trials, initial phenolic compounds concentration and initial color content were determined after sterilization.

### 4.4. Repeatability—Statistical Treatment

**Figure 5:**
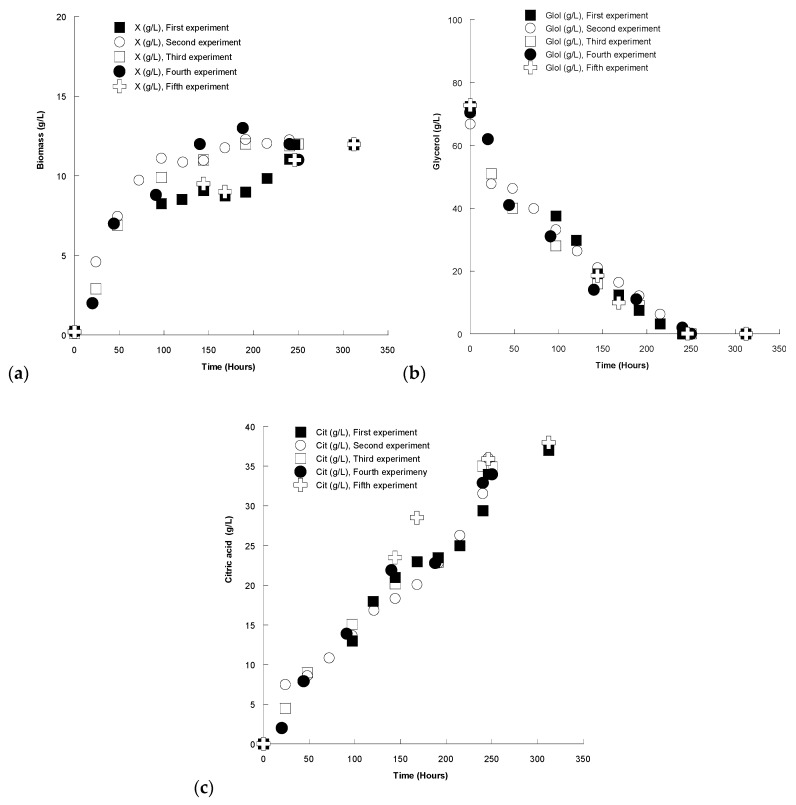
Kinetics of (**a**) biomass production, (**b**) remaining glycerol and (**c**) produced citric acid during growth of *Yarrowia lipolytica* strain ACA-DC 5029 on media with ~70 g/L initial crude glycerol concentration (no OMW addition) Five experiments under the same culture conditions with different inocula were performed. Culture conditions as described in Table 1.

In order to ascertain the validity of the experimental findings, additional experiments were conducted. Specifically, 5 different shake-flask cultures of *Y. lipolytica* (blank experiments—no OMW added into the medium) in which initial glycerol had been adjusted at ~70 g/L were carried out at initial pH = 6.0, pH ranging between 5.0 and 6.0 throughout culture, agitation at 180 ± 5 rpm and T = 28 ± 1 °C. The kinetics regarding biomass production (Figure 5a), glycerol consumption (Figure 5b) and citric acid production (Figure 5c) are presented in the Figure below.

The repeatability of the experiments as regards biomass biosynthesis, citric acid production and glycerol assimilation seems satisfactory. Moreover, flasks for the five different experiments were removed from the shaker and analyses were carried out at t = 142 ± 1 h after inoculation and t = 242 ± 2 h after inoculation. The obtained result for t = 142 ± 1 h after inoculation and for the 5 individual cultures showed the following feature: for biomass production, maximum and minimum values were 9.08 and 11.99 g/L respectively, with a mean value of 10.51 g/L, an obtained standard deviation of 1.196, a standard error of 0.535 and a variance of 1.429. For the case of remaining glycerol, maximum and minimum values were 21.10 and 14.00 g/L respectively, with a mean value of 17.75 g/L, an obtained standard deviation of 2.775, a standard error of 1.241 and a variance of 7.699. Concerning produced citric acid, maximum and minimum values were 23.50 and 18.34 g/L respectively, with a mean value of 20.99 g/L, an obtained standard deviation of 1.922, a standard error of 0.859 and a variance of 3.694. 

The obtained result for t = 242 ± 2 h after inoculation and for five individual cultures the following feature: for biomass production, maximum and minimum values were 12.26 and 11.00 g/L respectively, with a mean value of 11.64 g/L, an obtained standard deviation of 0.581, a standard error of 0.260 and a variance of 0.338. For remaining glycerol, maximum and minimum values were 2.01 and 0.00 g/L respectively, with a mean value of 0.62 g/L, an obtained standard deviation of 0.883, a standard error of 0.395 and a variance of 0.779. Concerning produced citric acid, maximum and minimum values were 35.00 and 29.40 g/L respectively, with a mean value of 32.95 g/L, an obtained standard deviation of 2.621, a standard error of 1.172 and a variance of 6.867.

## 5. Conclusions

*Y. lipolytica* strain ACA-DC 5029 was tested for its ability to grow on blends of crude glycerol and OMW. Even though this food industry-derived waste stream includes in its composition microbial inhibitors, the yeast presented satisfactory growth, suggesting that OMW could be characterized as a “lipogenic” medium. Additionally, citric acid production was not affected by the addition of OMW to the blends, indicating that it could partially or in some cases totally substitute tap water for concentrated glycerol dilution in the bioprocess proposed; thus, offering an attractive alternative in a potential industrial (large-scale) application. Partial removal of phenolic compounds and of color of the media occurred. Moreover, the yeast strain presented the ability to grow on media with ascending concentration of crude glycerol and produce significant amounts of citric acid and erythritol. This fact is of great importance as the surplus of crude glycerol deriving not only from biodiesel industry but also from oleochemical and food industry activities will negatively affect glycerol price. The strain studied, could be considered as a candidate for the biotransformation of crude biodiesel-derived glycerol and blends of OMW and crude glycerol towards the production of chemical compounds of added value. 

## Figures and Tables

**Table 1:** Kinetic data of *Yarrowia lipolytica* strain ACA-DC 5029 cultivated on crude glycerol with initial concentration of ~70 g/L, blended with olive mill wastewaters in various amounts.

Initial Phenolics (g/L)	Hours	X (g/L)	L (g/L)	Glol_cons_ (g/L)	Cit (g/L)	Man (g/L)	Ara (g/L)	Ery (g/L)	Y_X/Glol_ (g/g)	Y_L/X_ % (*w*/*w*)	Y_Cit/Glol_ (g/g)	Y_Man/Glol_ (g/g)	Y_Ara/Glol_ (g/g)	Y_Ery/Glol_ (g/g)
0.0	191 ^f^	8.98	0.60	64.9	17.3	9.9	3.1	14.9	0.14	6.7	0.27	0.15	0.05	0.23
	240 ^d^	11.04	0.76	72.4	29.4	10.1	2.9	14.5	0.15	6.9	0.41	0.14	0.04	0.20
	246 ^a,b,e^	11.96	1.27	72.4	34.0	8.1	3.2	9.1	0.17	10.6	0.47	0.11	0.04	0.13
	312 ^c^	11.95	0.62	72.4	42.5	6.8	3.0	4.1	0.17	5.2	0.59	0.09	0.04	0.06
~1.0	73 ^b^	9.26	1.65	25.4	2.6	3.4	0.8	3.7	0.36	17.8	0.10	0.13	0.03	0.15
	124 ^f^	10.48	1.25	50.6	12.0	6.6	2.0	8.9	0.21	11.9	0.24	0.13	0.04	0.18
	193 ^a^	11.56	0.63	66.2	25.1	8.8	2.0	7.8	0.17	5.5	0.38	0.13	0.03	0.12
	241 ^c,d,e^	11.41	0.28	70.0	28.8	10.9	2.5	5.6	0.16	2.4	0.41	0.16	0.04	0.08
~2.0	192 ^b^	9.74	1.95	48.8	9.5	3.9	1.2	13.1	0.20	20.0	0.19	0.08	0.03	0.27
	242 ^a,c,d,e,f^	11.58	1.60	70.0	31.5	5.3	2.0	13.5	0.17	13.8	0.45	0.08	0.03	0.19
~3.5	140 ^b,d,f^	8.30	1.32	61.4	26.2	13.1	1.2	2.4	0.14	15.9	0.43	0.21	0.02	0.04
	240 ^a,c,e^	8.72	0.81	68.1	37.4	10.1	3.1	0.0	0.13	9.3	0.55	0.15	0.05	0.00

Representations (in g/L) of biomass (X), cellular lipid (L), consumed substrate (Glol_cons_), citric acid (Cit), mannitol (Man), arabitol (Ara) erythritol (Ery), % (*w*/*w*) yield lipid in biomass (Y_L/X_) and (in g/g) respective yield of biomass on glycerol consumed (Y_X/Glol_), conversion yield of citric acid produced per glycerol consumed (Y_Cit/Glol_), conversion yield of mannitol produced per glycerol consumed (Y_Man/Glol_), conversion yield of arabitol produced per glycerol consumed (Y_Ara/Glol_) and conversion yield of erythritol produced per glycerol consumed (Y_Ery/Glol_) at different fermentation hours. ^a^ when X_max_ concentration achieved. ^b^ when L_max_ concentration achieved. ^c^ when Cit_max_ concentration achieved. ^d^ when Man_max_ concentration achieved. ^e^ when Ara_max_ concentration achieved. ^f^ when Ery_max_ concentration achieved. Culture conditions: growth on aseptic 250 mL flasks at 180 ± 5 rpm, Peptone = 1.0 g/L, yeast extract = 1.0 g/L, pH ranging between 5.0 and 6.0, DOT > 20% *v*/*v*, incubation temperature T = 28 °C. Each point is the mean value of two independent measurements.

**Table 2:** Fatty acid composition in the total cellular lipid (% *w*/*w*) of *Yarrowia lipolytica* strain ACA-DC 5029 cultivated on crude glycerol with initial concentration of ~70 g/L, blended with olive mill wastewaters in various amounts.

Initial Phenolics (g/L)	Time (h)	C16:0	^Δ9^C16:1	C18:0	^Δ9^C18:1	^Δ9,12^C18:2	UI
0.0	72	21.9	9.9	12.4	43.3	12.3	0.778
	144	16.9	13.9	6.5	48.3	14.2	0.906
	216	17.1	13.5	8.1	46.6	14.4	0.889
~1.0	48	19.8	10.8	11.3	46.0	12.1	0.810
	145	16.0	11.4	8.0	50.9	13.7	0.897
	217	17.2	11.0	9.2	51.1	11.5	0.851
~2.0	48	20.1	10.7	8.1	48.4	12.7	0.845
	144	11.5	4.5	5.4	61.3	17.3	1.004
	216	16.2	13.6	7.0	50.4	12.8	0.896
~3.5	45	20.0	4.4	6.6	53.9	15.1	0.885
	140	18.3	7.8	7.4	50.6	15.9	0.902
	209	16.7	3.0	6.8	56.4	17.1	0.937

UI = [% monoene + 2 (%diene) + 3 (%triene)]/100. Culture conditions as described in Table 1. Each experimental point is the mean value of two determinations (SE < 10%).

**Table 3:** Kinetic data of *Yarrowia lipolytica* strain ACA-DC 5029 cultivated on crude glycerol with initial concentrations of ~70 g/L, 120 g/L and of ~170 g/L.

Glol_0_ (g/L)	Hours	X (g/L)	L (g/L)	Glol_cons_ (g/L)	Cit (g/L)	Man (g/L)	Ara (g/L)	Ery (g/L)	Y_X/Glol_ (g/g)	Y_L/X_ % (*w*/*w*)	Y_Cit/Glol_ (g/g)	Y_Man/Glol_ (g/g)	Y_Ara/Glol_ (g/g)	Y_Ery/Glol_ (g/g)
~70.0	191 ^f^	8.98	0.60	64.9	17.3	9.9	3.1	14.9	0.14	6.7	0.27	0.15	0.05	0.23
	240 ^d^	11.04	0.76	72.4	29.4	10.1	2.9	14.5	0.15	6.9	0.41	0.14	0.04	0.20
	246 ^a,b,e^	11.96	1.27	72.4	34.0	8.1	3.2	9.1	0.17	10.6	0.47	0.11	0.04	0.13
	312 ^c^	11.95	0.62	72.4	42.5	6.8	3.0	4.1	0.17	5.2	0.59	0.09	0.04	0.06
~120.0	264 ^b,d,,e,f^	10.52	2.07	98.5	33.5	8.0	4.5	38.4	0.11	19.7	0.34	0.08	0.05	0.39
	384 ^a,c^	12.40	1.11	122.9	63.8	7.2	4.2	31.4	0.10	9.0	0.52	0.06	0.03	0.26
~170.0	408 ^b^	11.36	2.54	132.8	63.3	4.0	1.1	47.3	0.09	22.4	0.48	0.03	0.01	0.36
	480 ^a^	12.48	2.52	160.5	72.9	4.6	1.9	58.8	0.08	15.9	0.45	0.03	0.01	0.37
	528 ^c,d,e,f^	11.98	0.83	170.6	79.0	6.5	3.4	65.8	0.07	6.9	0.46	0.04	0.02	0.39

Representations (in g/L) of biomass (X), cellular lipid (L), consumed substrate (Glol_cons_), citric acid (Cit), mannitol (Man), arabitol (Ara) erythritol (Ery), % (*w*/*w*) yield lipid in biomass (Y_L/X_) and (in g/g) respective yield of biomass on glycerol consumed (Y_X/Glol_), conversion yield of citric acid produced per glycerol consumed (Y_Cit/Glol_), conversion yield of mannitol produced per glycerol consumed (Y_Man/Glol_), conversion yield of arabitol produced per glycerol consumed (Y_Ara/Glol_) and conversion yield of erythritol produced per glycerol consumed (Y_Ery/Glol_) at different fermentation hours. ^a^ when X_max_ concentration achieved. ^b^ when L_max_ concentration achieved. ^c^ when Cit_max_ concentration achieved. ^d^ when Man_max_ concentration achieved. ^e^ when Ara_max_ concentration achieved. ^f^ when Ery_max_ concentration achieved. Culture conditions as described in Table 1. Each point is the mean value of two independent measurements.

**Table 4:** Fatty acid composition in the total cellular lipid (% *w*/*w*) of Yarrowia lipolytica strain ACA-DC 5029 when cultivated on crude glycerol with initial concentration of ~70 g/L, 120 g/L and of ~170 g/L.

Glol_0_ (g/L)	Time (h)	C16:0	^Δ9^C16:1	C18:0	^Δ9^C18:1	^Δ9,12^C18:2	UI
~70	72	21.9	9.9	12.4	43.3	12.3	0.778
	144	16.9	13.9	6.5	48.3	14.2	0.906
	216	17.1	13.5	8.1	46.6	14.4	0.889
~120	48	15.3	16.3	5.0	50.1	13.2	0.928
	120	15.7	22.3	4.8	47.7	9.3	0.886
	216	16.4	24.1	4.2	48.1	7.1	0.864
	288	14.4	23.4	4.3	49.5	8.3	0.895
	336	16.9	29.9	5.2	63.0	11.8	1.165
	456	18.0	17.5	6.2	6.2	52.0	1.278
~170	72	36.1	14.8	16.2	26.6	6.3	0.540
	168	18.2	28.9	5.0	41.9	6.1	0.830
	264	14.7	22.5	2.9	52.6	7.3	0.897
	360	12.9	32.0	2.9	45.9	6.4	0.906
	432	12.2	31.7	3.3	45.1	7.7	0.922
	504	14.1	28.7	5.0	44.8	7.3	0.881

UI = [% monoene + 2 (%diene) + 3 (%triene)]/100. Culture conditions as described in Table 1. Each experimental point is the mean value of two determinations (SE < 10%).

## Nomenclature

OMW	Olive mill wastewaters
X (g/L)	Biomass
Glol (g/L)	Glycerol
Ara (g/L)	Arabitol
Ery (g/L)	Erythritol
Man (g/L)	Mannitol
Cit (g/L)	Citric acid
L (g/L)	Total lipid
Ph (g/L)	Phenolic compounds
Y_X/Glol_ (g formed per g of glycerol consumed)	Biomass yield on glycerol consumed
Y_Ara/Glol_ (g formed per g of glycerol consumed)	Arabitol yield on glycerol consumed
Y_Ery/Glol_ (g formed per g of glycerol consumed)	Erythritol yield on glycerol consumed
Y_Man/Glol_ (g formed per g of glycerol consumed)	Mannitol yield on glycerol consumed
Y_Cit/Glol_ (g formed per g of glycerol consumed)	Citric acid yield on glycerol consumed
Y_L/X_ (% *w*/*w*)	Total lipid on biomass
UI	Unsaturation index
FA	Fatty acid
IPS (g formed per g of biomass formed; % *w*/*w*)	Endopolysaccharides
Subscripts 0, cons and max indicate the initial, consumed and maximum quantity of the components in the kinetics performed	
